# Pulmonary Embolism Originating From a Hepatic Hydatid Cyst: A Case Report

**DOI:** 10.7759/cureus.80990

**Published:** 2025-03-22

**Authors:** Abdelkader Boukharta, Khalid Bouti, Sanaa Hammi

**Affiliations:** 1 Department of Pulmonology, Mohammed VI University Hospital, Tangier, MAR; 2 Laboratory of Life and Health Sciences, Faculty of Medicine and Pharmacy of Tangier, Abdelmalek Essaâdi University, Tangier, MAR

**Keywords:** complications, echinococcosis, hydatid cyst, pulmonary embolism, zoonotic diseases

## Abstract

Spontaneous pulmonary embolism due to hydatid disease is an extremely rare complication. Here, we present a patient case involving multiple cystic hydatid lesions within the lung parenchyma and liver. A 32-year-old woman was admitted with acute respiratory failure and chest pain. Chest radiographs revealed a solitary oval opacity in the upper zone of the right lung. CT angiography was performed, showing a concomitant lung and liver hydatid cyst that had fistulized into the inferior vena cava, leading to massive bilateral hydatid pulmonary embolism, echinococcosis serologic testing was positive, pulmonary embolism was attributed to the hydatid cyst. Pulmonary hydatid embolism is a rare but serious complication of liver hydatid cysts. This diagnosis should be considered, particularly in endemic areas of this zoonotic disease.

## Introduction

Hydatid cysts in humans occur as a result of infection by the larval stages of Echinococcus granulosus. The highest prevalence of cystic echinococcosis in human and animal hosts is observed in temperate regions, including southern South America, the entire Mediterranean littoral, southern and central parts of the former Soviet Union, Central Asia, China, Australia, and parts of Africa [1,2].

The liver and lungs are the principal sites of involvement [3]; however, hydatid cysts can occur in any organ. Pulmonary embolism caused by a hydatid cyst is considered a rare entity and can lead to cor pulmonale and chronic respiratory failure [4].

Hepatic hydatid cysts can rupture into the portal vein, resulting in bilateral pulmonary artery embolism. Daughter vesicles migrate from the right heart to the pulmonary arteries [5].

## Case presentation

We present a rare case of pulmonary embolism caused by a hydatid cyst. A 32-year-old Moroccan woman, born in a rural area, non-smoker, with no comorbidities, had a history of contact with dogs and no risk factors for venous thromboembolism.

The patient was admitted to the emergency department with acute respiratory failure and chest pain. She reported having had a productive cough for six months, with occasional episodes of hemoptysis in small quantities. One day before admission, she experienced a generalized itching (grade 1 anaphylactic reaction) but had no other general symptoms.

Physical examination is as follows: SpO₂ on room air, 84%; on supplemental O₂ (8 L/min), 95%. Clinical examination revealed abdominal tenderness in the right upper quadrant.

Laboratory findings are given in Table 1.

**Table 1:** Biological test results

Parameter	Result	Reference Values
Leukocytes	9.11 x 10⁹/µL	4-10 x 10⁹/µL
Neutrophils	5.01 x 10⁹/µL (55.0%)	1.5-7.0 x 10⁹/µL (50-70%)
Lymphocytes	1.57 x 10⁹/µL (17.2%)	1-4 x 10⁹/µL (20-40%)
Monocytes	0.31 x 10⁹/µL (3.4%)	0.3-1 x 10⁹/µL (3-12%)
Eosinophils	2.15 x 10⁹/µL (23.6%)	0.1-0.4 x 10⁹/µL (0.5-5%)
Basophils	0.07 x 10⁹/µL (0.8%)	0-0.1 x 10⁹/µL (0-1%)
Red Blood Cells (RBCs)	4.74 x 10¹²/µL	4-5.2 x 10¹²/µL
Hemoglobin (HGB)	14.2 g/dL	12-16 g/dL
Hematocrit (HCT)	41.9%	37-47%
Mean Corpuscular Volume (MCV)	88.3 fL	79-99 fL
Mean Corpuscular Hemoglobin (MCH)	29.8 pg	27-32 pg
Platelets (PLT)	238 x 10⁹/µL	150-400 x 10⁹/µL
Mean Platelet Volume (MPV)	9.2 fL	6.5-12 fL
Platelet Distribution Width (PDW)	16.7	9-17
C-Reactive Protein	52.40 mg/L	0-6 mg/L
Urea	0.17 g/L	0.15-0.45 g/L
Serum Creatinine	6.50 mg/L	6-11 mg/L
Aspartate Aminotransferase (AST/SGOT)	15.11 U/L	< 31 U/L
Alanine Aminotransferase (ALT/SGPT)	20.92 U/L	< 34 U/L
High-Sensitivity Troponin I	99 ng/L	< 10 ng/L: No myocardial injury, 10-80 ng/L: Myocardial injury, > 80 ng/L: Myocardial infarction
Hydatid Serology (Echinococcosis) *Chemiluminescence assay*	Positive (1/2560)	< 1/160: Non-significant, 1/160: Doubtful, > 1/160: Significant reaction indicating evolving hydatidosis
D-dimer	649	<500
Blood Group	O	-
Rh Factor	Positive	-

Radiological findings

**Figure 1:**
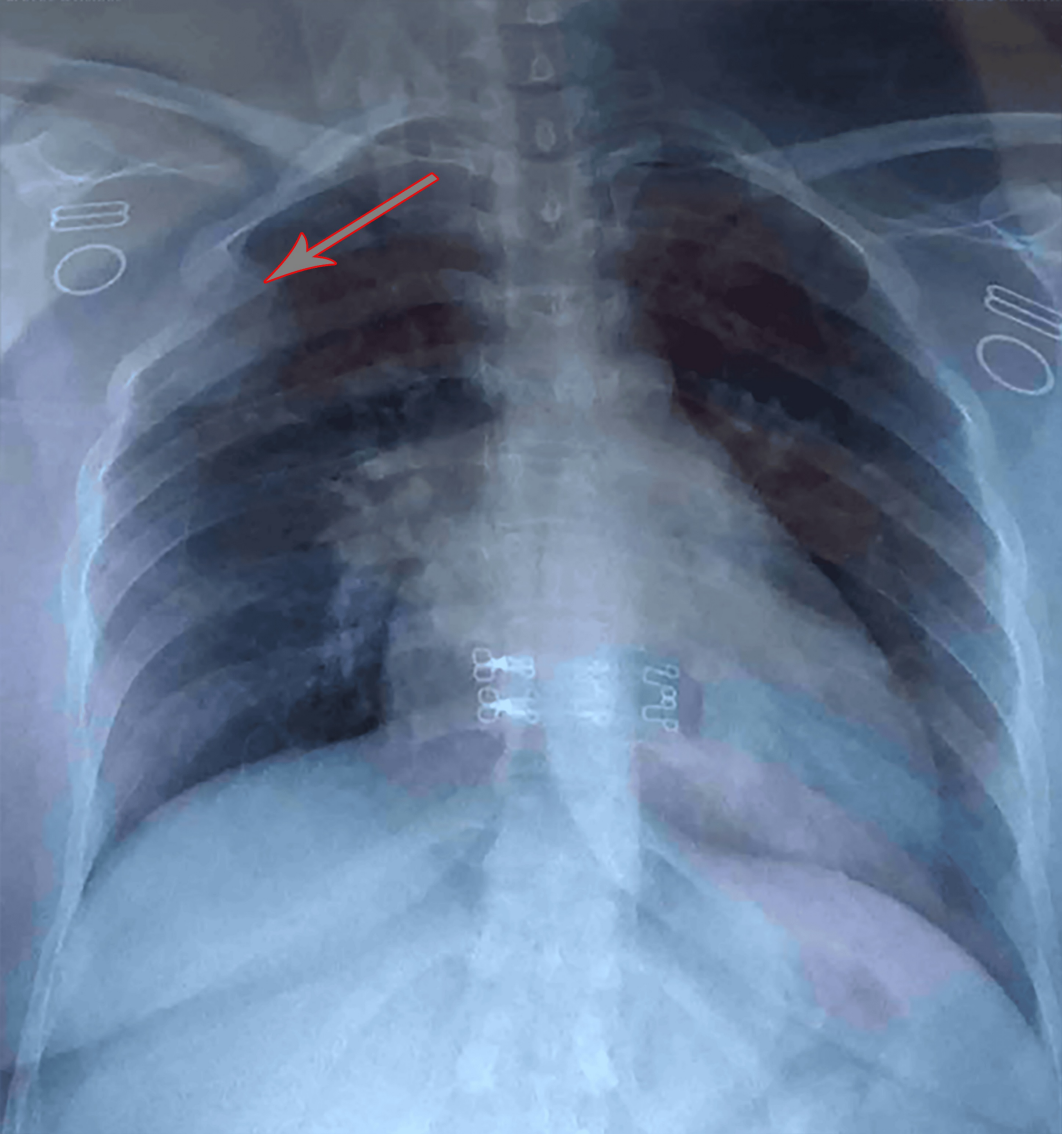
Chest X-ray shows the lung hydatid cyst (arrow).

Chest X-ray showed a homogeneous oval opacity in the upper lobe of the right lung (Figure 1).

**Figure 2:**
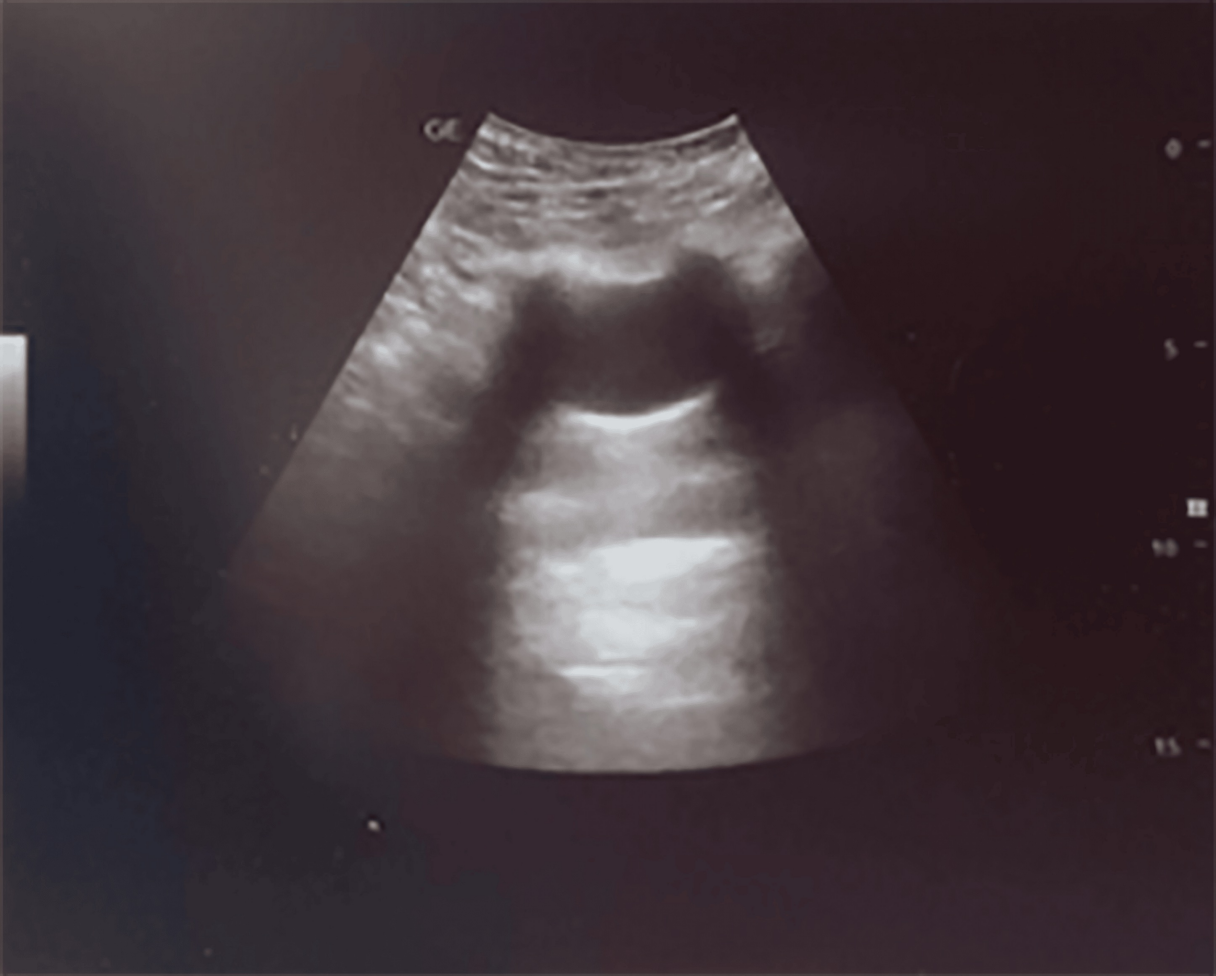
Chest ultrasound of pulmonary cyst.

Chest ultrasound revealed a simple cyst in the right upper zone, presenting as an anechoic, well-defined structure with a thin, regular wall and posterior acoustic enhancement (Figure 2).

**Figure 3:**
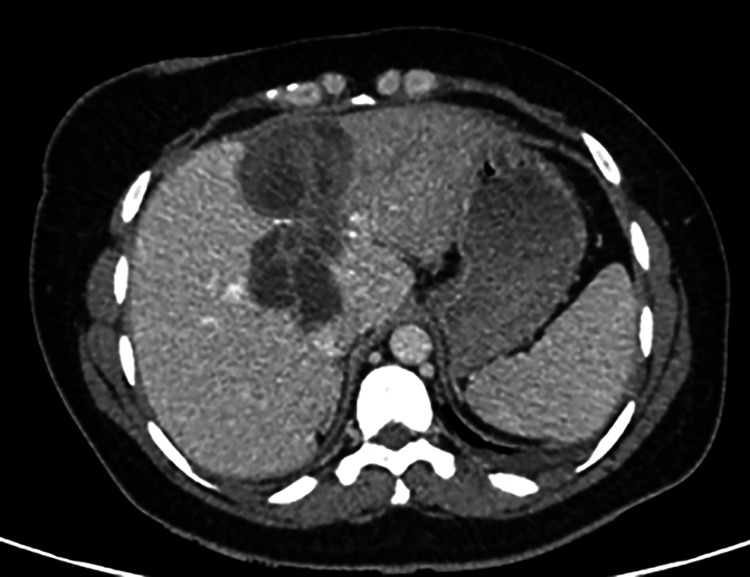
CT scan detected two hydatid cysts of the liver.

Abdominal ultrasound and abdominal CT scan detected two hydatid cysts of the liver classified as type III and IV, according to Gharbi classification (Figure 3).

Echocardiography showed a dilatation of the right ventricle and paradoxical movement of the interventricular septum.

**Figure 4:**
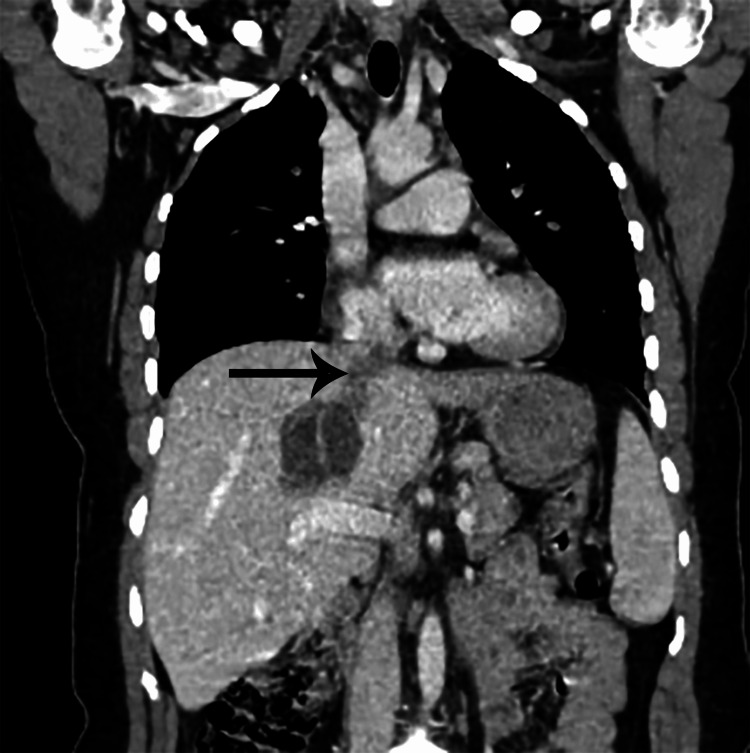
Liver hydatid cyst fistulized into inferior vena cava.

**Figure 5:**
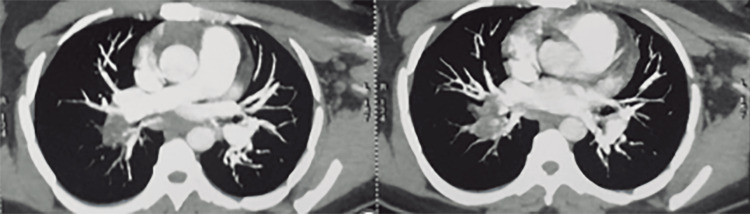
Proximal massive pulmonary embolism.

CT-angiography was performed and showed concomitant lung and liver hydatid cyst fistulized into the inferior vena cava, resulting in a hydatid bilateral massive pulmonary embolism (Figure 4 and Figure 5).

During hospitalization, the patient stabilized on respiratory and hemodynamic status, the hemoptysis was controlled with tranexamic acid, and she received anticoagulation (Enoxaparin) in order to block and prevent the constitution of thrombus. After seven days, the patient was stabilized on respiratory symptoms (SpO_2_ 98%, respiratory rate 20 cpm). Due to the worsening chest pain, we reassessed the pulmonary embolism with a follow-up angiography, which revealed radiological progression. Echocardiography was subsequently performed and showed normal findings. After the clinical stabilization of the patient, we conducted a multidisciplinary discussion to evaluate the indication for surgical intervention. It was concluded that clamping of the inferior vena cava and neutralizing the fistula between the inferior vena cava and hepatic hydatid cyst was necessary. The postoperative course was uneventful.

Three months after the surgery, the patient reported two episodes of hemoptysis. She was treated with albendazole for six months following the surgical intervention.

**Figure 6:**
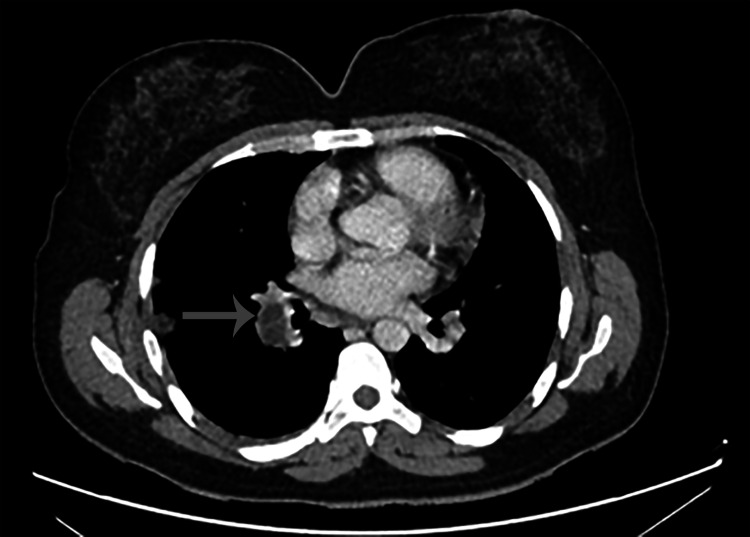
One-year follow-up CT scan.

The patient received albendazole for six months after the surgical intervention. She is still alive after one year and six months after surgical intervention. The patient shows an improvement in dyspnea, but she continues to have dyspnea classified as grade 1 according to the modified Medical Research Council (mMRC) scale. The echocardiography did not show any signs suggestive of pulmonary hypertension. A follow-up CT scan was performed one year later, revealing the persistence of hypodense intraluminal content within the branches of the pulmonary artery (Figure 6).

## Discussion

The clinical presentation of pulmonary hydatid embolism is non-specific. However, it is necessary to assess the patient’s history, and the presence of systemic reactions, especially in the initial phase of cyst rupture. The symptoms range from simple discomfort associated with signs of minor allergy such as skin rash to anaphylactic shock. The risk of sudden death is high during this phase due to massive pulmonary embolism, anaphylactic shock, or stenosis of a valve orifice. The sites of hydatid cyst formation within human tissues are the liver (60-75%), the lung (15-25%), and the remaining parts of the body (10-15%) [6]. Pulmonary hydatid embolism can occur classically if its location is in the right cavities. Hydatid embolism secondary to fistulization into the inferior vena cava allows daughter vesicles to migrate through the venous circulation into the right atrium, right ventricle, and finally the pulmonary artery which contributes, therefore, to a rarely described liver cyst. Three types of cyst rupture are recognized: contained, communicating and direct. Pulmonary hydatid embolism has been described in the literature under three forms: (i) acute fatal embolism, (ii) sub-acute embolism resulting in pulmonary hypertension and death in less than one year, and (iii) chronic pulmonary hypertension [6,7]. Our patient was admitted with acute cor pulmonale. Early diagnosis of hydatid pulmonary embolism and treatment may give a chance to escape the threatening stage and an improved outcome, as in the case presented. In a Moroccan case series of three patients in which the surgical treatment was rejected due to the significant operative risk, a medical treatment with albendazole for three to six months was prescribed [8].

By comparing different case series, it becomes evident that there is no standardized treatment protocol for hydatid pulmonary embolism. Early diagnosis and a multidisciplinary approach are crucial for optimizing patient outcomes (Table 2).

**Table 2:** Reported cases of pulmonary embolism due to hepatic hydatid disease.

Study	Year	Number of Cases	Patient Demographics	Clinical Presentation	Diagnostic Methods	Treatment Approaches	Outcomes
Aili et al. [9]	2021	1	58-year-old woman	Chest and back pain, shortness of breath, hemoptysis	CT pulmonary angiography	Multidisciplinary management	Not specified
Yuan et al. [5]	2015	1	70-year-old man	Severe cough, hemoptysis post hepatic hydatid cyst surgery	Chest radiographs, CT, MRI, serologic testing	Not specified	Not specified
Mezgar et al. [10]	2018	1	56-year-old woman	Posttraumatic abdominal pain	Chest radiographs, CT scan	Not specified	Not specified
Abid et al. [11]	2011	1	16-year-old boy	Exercise-induced dyspnea, hemoptysis	Chest X-ray, ECG, imaging	Surgical removal	Uneventful recovery

## Conclusions

Spontaneous pulmonary embolism caused by hydatid disease is an exceptionally rare complication. It is essential to consider this diagnosis, especially in regions where this zoonotic disease is endemic.

The primary approach to control remains prevention, which includes deworming dogs, ensuring proper slaughterhouse hygiene, and raising public awareness through education programs.
